# Correction and Republication: New COVID-19 Cases and Hospitalizations Among Adults, by Vaccination Status — New York, May 3–July 25, 2021

**DOI:** 10.15585/mmwr.mm7037a6

**Published:** 2021-09-17

On, August 18, 2021, *MMWR* published “New COVID-19 Cases and Hospitalizations Among Adults, by Vaccination Status — New York, May 3–July 25, 2021” (*1*). On August 25, 2021, the authors informed *MMWR* that some analyses were inaccurate because vaccination records of persons with a birth date between two vaccination dates could be counted as two distinct persons with different ages. This resulted in an artificial inflation of the population of partially vaccinated persons, which in turn affected the number of unvaccinated persons because that number is estimated as the total population size minus the fully vaccinated and the partially vaccinated groups. Programming code was adjusted to address this issue as well as three uncommon issues that had a relatively minor impact on findings. First, unvaccinated persons who received positive test results for SARS-CoV-2 who subsequently received a first vaccination dose were not always counted towards the tally of unvaccinated COVID-19 cases. Second, persons who received additional doses before such doses were authorized had their date of full vaccination assigned based on final dose date, rather than series completion date. Third, persons who received doses in both New York City and the other areas of New York required additional deduplication. Using current data from the continuously updated surveillance databases, the authors have corrected the *MMWR* report accordingly and confirmed that the interpretation and the conclusions of the original report were not affected by these changes (the updated results are highly similar to those of the primary analysis and sensitivity analyses as reported in the original paper). *MMWR* has republished the report (*2*), which includes the original report with clearly marked corrections in supplementary materials.
